# Screening of malaria infections in human blood samples with varying parasite densities and anaemic conditions using AI-Powered mid-infrared spectroscopy

**DOI:** 10.1186/s12936-024-05011-z

**Published:** 2024-06-17

**Authors:** Issa H. Mshani, Frank M. Jackson, Rehema Y. Mwanga, Prisca A. Kweyamba, Emmanuel P. Mwanga, Mgeni M. Tambwe, Lorenz M. Hofer, Doreen J. Siria, Mario González-Jiménez, Klaas Wynne, Sarah J. Moore, Fredros Okumu, Simon A. Babayan, Francesco Baldini

**Affiliations:** 1 Environmental Health, and Ecological Sciences Department, Ifakara Health Institute, Morogoro, United Republic of Tanzania; 2 School of Biodiversity, One Health and Veterinary Medicine, The University of Glasgow, Glasgow, UK; 3 Swiss Tropical and Public Health Institute, Kreuzstrasse 2, 4123 Allschwil, Switzerland; 4 University of Basel, Petersplatz 1, 4001 Basel, Switzerland; 5 School of Chemistry, The University of Glasgow, Glasgow, G128QQ UK; 6 School of Life Sciences and Biotechnology, Nelson Mandela African Institution of Science and Technology, Arusha, United Republic of Tanzania; 7 School of Public Health, The University of the Witwatersrand, Park Town, Johannesburg, South Africa

## Abstract

**Background:**

Effective testing for malaria, including the detection of infections at very low densities, is vital for the successful elimination of the disease. Unfortunately, existing methods are either inexpensive but poorly sensitive or sensitive but costly. Recent studies have shown that mid-infrared spectroscopy coupled with machine learning (MIRs-ML) has potential for rapidly detecting malaria infections but requires further evaluation on diverse samples representative of natural infections in endemic areas. The aim of this study was, therefore, to demonstrate a simple AI-powered, reagent-free, and user-friendly approach that uses mid-infrared spectra from dried blood spots to accurately detect malaria infections across varying parasite densities and anaemic conditions.

**Methods:**

*Plasmodium falciparum* strains NF54 and FCR3 were cultured and mixed with blood from 70 malaria-free individuals to create various malaria parasitaemia and anaemic conditions. Blood dilutions produced three haematocrit ratios (50%, 25%, 12.5%) and five parasitaemia levels (6%, 0.1%, 0.002%, 0.00003%, 0%). Dried blood spots were prepared on Whatman^™^ filter papers and scanned using attenuated total reflection-Fourier Transform Infrared (ATR-FTIR) for machine-learning analysis. Three classifiers were trained on an 80%/20% split of 4655 spectra: (I) high contrast (6% parasitaemia vs. negative), (II) low contrast (0.00003% vs. negative) and (III) all concentrations (all positive levels vs. negative). The classifiers were validated with unseen datasets to detect malaria at various parasitaemia levels and anaemic conditions. Additionally, these classifiers were tested on samples from a population survey in malaria-endemic villages of southeastern Tanzania.

**Results:**

The AI classifiers attained over 90% accuracy in detecting malaria infections as low as one parasite per microlitre of blood, a sensitivity unattainable by conventional RDTs and microscopy. These laboratory-developed classifiers seamlessly transitioned to field applicability, achieving over 80% accuracy in predicting natural *P. falciparum* infections in blood samples collected during the field survey. Crucially, the performance remained unaffected by various levels of anaemia, a common complication in malaria patients.

**Conclusion:**

These findings suggest that the AI-driven mid-infrared spectroscopy approach holds promise as a simplified, sensitive and cost-effective method for malaria screening, consistently performing well despite variations in parasite densities and anaemic conditions. The technique simply involves scanning dried blood spots with a desktop mid-infrared scanner and analysing the spectra using pre-trained AI classifiers, making it readily adaptable to field conditions in low-resource settings. In this study, the approach was successfully adapted to field use, effectively predicting natural malaria infections in blood samples from a population-level survey in Tanzania. With additional field trials and validation, this technique could significantly enhance malaria surveillance and contribute to accelerating malaria elimination efforts.

**Supplementary Information:**

The online version contains supplementary material available at 10.1186/s12936-024-05011-z.

## Background

Malaria control has made significant progress in recent decades but there are still an estimated 600,000 deaths and 250 million cases annually, most of these in sub-Saharan Africa [1]. To accelerate elimination efforts, effective strategies are required, both for control and for surveillance. There is an urgent need for simple, scalable and low-cost methods for monitoring key malaria metrics, so as to establish prevailing risk, evaluate impacts of control measures and assess overall progress against malaria. The World Health Organization (WHO) underscores the need for evidence-based and context-specific control strategies—which require sensitive, rapid, and affordable screening tools that are deployable at scale even in low-income or remote settings [2]. This includes the need for accurate detection of malaria infections—both at clinical points of care settings and in population surveys.

While rapid diagnostic tests (RDTs), microscopy, and polymerase chain reactions (PCR) have played a crucial role in the diagnosis and management of malaria across endemic and elimination situations [3], these approaches still have major limitations in most settings. For example, RDTs have transformed malaria case management across Africa due to their practicality and affordability [4], yet studies show that as many as half of children in some poor communities still lack access to these tests [5, 6]. Their effectiveness is also increasingly threatened by deletions on the histidine-rich protein 2 and 3 (HRP II and III) genes, which code for the protein targets of most *Plasmodium falciparum* RDTs [7–9]. Additionally, both microscopy and RDTs have poor sensitivity when parasite densities fall below 50–100 parasites/μL of blood [3, 6, 10–12]; and can miss significant fractions of sub-microscopic infections, which can contribute 20–50% of all human-to-mosquito transmission in low endemicity settings [13]. From an operational perspective, microscopy requires electrical power sources, can take as long as 30 min to run, is prone to subjective interpretations of results and can be expensive due to personnel costs and reagents [14]. Unfortunately, even the highly sensitive techniques, such as PCR, enzyme-linked immunosorbent assays (ELISA), and loop-mediated isothermal amplification (LAMP), which detect as few as 5 parasites/μL of blood, with greater accuracy, are expensive, and require highly skilled workers.

In recent years, infrared spectroscopy (IR) in both the near (NIR) and mid (MIR) infrared ranges has shown substantial promise for monitoring key entomological and parasitological indicators of malaria—including detecting malaria infections in human blood [15–17]. The technology is rapid, robust, reagent-free, and requires minimum skills to operate [15, 18]. Infrared spectroscopy involves shining infrared light through biological specimens to infer their biochemical composition, which can be analysed using different mathematical techniques to distinguish meaningful traits, such as infection status, age, and species. Given the complexities of assigning spectroscopic bands for interpreting patterns associated with malaria infections, recent studies have been integrating spectroscopy with either multivariate analysis or machine learning (ML) approaches to more effectively interpret the key biological traits [15, 19, 20]. Infrared spectroscopy combined with machine learning (IR-ML), outperforms the traditional analyses in detecting malaria by efficiently deciphering complex, non-linear, multi-correlated spectral signals and exhibiting high accuracy [15, 18]. For example, during the development cycle, malaria parasites generate distinctive biomarkers, which are evident on infrared spectra and can be used to identify infected individuals [20–23]. Further, models have been reported to achieve over 90% precision in differentiating infected from uninfected blood samples [16, 23].

Despite IR-ML techniques demonstrating potential in identifying malaria infections in whole blood or isolated red blood cells (RBCs), critical gaps remain to be addressed, one of which is the need to evaluate the lower limits of parasite detections and quantifications [15]. One study has shown that MIR coupled with multivariate statistics could detect low density malaria infections at < 1–5 parasites/µL of blood in methanol-fixed RBCs [21]. However, no tests have been done on the actual detection thresholds critical for applications of IR-ML in either point-of-care tests or population surveys using whole blood specimens.

Another key gap is the need to identify and estimate the impacts of confounding factors. In areas with high malaria transmission, infected individuals often exhibit decreased haemoglobin concentrations, other parasitic infections, nutritional deficiencies or other concomitant factors [24–26]; all of which may compromise tools such as IR-ML that are designed to detect biochemical changes resulting from malaria infections. Indeed, previous MIR-ML models trained for malaria detection may have been influenced by the association of co-variates (e.g., anaemia and immune response to parasites) rather than true parasite signals [16]. Immunological variations from persistent asymptomatic infections may also affect performance of IR-ML results for malaria detection, in ways not detectable in studies that use blood bank samples from limited numbers of volunteers. ML algorithms should therefore consider these variabilities, e.g. by incorporating varying anaemic, nutritional or immunologic conditions, but also train models with different parasite ‘contrasts’, to disentangle true infections more effectively from these noise signals.

The aim of this current study was therefore to assess the lower limit of malaria parasite detection on dried blood spots (DBS) using MIR-ML approaches, investigate the impact of anaemic conditions on detecting malaria infections and demonstrate the best ML training approaches, whether utilizing high, low, or combined parasite concentrations, and finally test them on spectra of actual malaria-infected patients. The DBS were generated using whole blood from 70 malaria-free volunteers to capture immunological variations, and were spiked with cultured ring-stage *P. falciparum* at various parasitaemia and haematocrit ratios to mimic anaemia. ML algorithms were then trained on MIR spectra acquired from laboratory-generated DBS, where specific malaria parasite signals could be learned. MIRs-ML, trained with high-contrast parasite concentrations against negatives, demonstrated a detection accuracy of over 90% in laboratory tests across various parasitaemia levels; in field-collected samples, the accuracy consistently remained above 80%.

## Methods

### Malaria parasite cultures

Sample collection and parasite cultures were completed at Ifakara Health Institute’s laboratories in Bagamoyo, Tanzania using adapted protocols with slight adjustments [27, 28]. Group O+ blood was obtained from four malaria-free volunteers, and kept in tubes containing the anticoagulant, ethylenediaminetetraacetic acid (EDTA) for continuous culturing of *P. falciparum* strains NF54 and FCR3. The blood was centrifuged at 2500 rpm at 24 °C for 10 min to obtain RBCs. The RBCs were then washed, diluted to 50% haematocrit with uncomplemented media, namely Roswell Park Memorial Institute (RPMI) 1640 media supplemented with hypoxanthine, neomycin and 4-(2-hydroxyethyl)-1-piperazineethanesulfonic acid (HEPES), and stored at 4 °C.

The washed RBCs were used to culture *P. falciparum *in vitro for up to 7 days. The asexual malaria parasites were grown in uninfected washed O+ RBCs as host cells at 5% haematocrit, maintained in RPMI-1640 medium supplemented with 25 mM HEPES, 100 µM hypoxanthine, neomycin, 5% Albumax II and 24 mM Sodium bicarbonate. The parasite culture was gassed with a mixture of 5% CO_2_, 3% O_2_, and 93% N_2_ and incubated at 37 °C. The culture was examined daily for parasitaemia estimation using field-stained (Hemacolor^®^ rapid staining) thin blood smears under a compound microscope in 10 fields. Two rounds of parasite synchronization was performed to ensure the remaining of only ring-stage *P. falciparum* [29]. The culture was kept until the ring-stage parasitaemia level reached > 6% and was used for experimental dilutions. Parallel malaria-free cultures with only media and O+ RBCs from the same volunteers were kept to create controls. The process was repeated for up to 11 batches, until 70 volunteers were recruited and their blood was diluted.

### Recruitment of malaria-free volunteers

Malaria-free individuals were recruited from tertiary-level colleges in Bagamoyo, eastern Tanzania, following sensitization meetings, during which the objectives, procedures, potential risks, and benefits of the study were explained. Participants who expressed interest were given a unique identity number, contacted by phone, and requested to provide informed consent. The consenting participants were screened for malaria parasites using RDTs by taking a finger-prick blood sample, followed by a confirmatory quantitative polymerase chain reaction (qPCR). Participants who tested negative for malaria were enrolled in the study, while those who tested positive were treated following Tanzania's malaria treatment guidelines and excluded from the study [30]. A total of 70 volunteer participants were recruited, all between 20 and 40 years old. A total of 40 mL venous blood was drawn in an EDTA tube from each participant and used for laboratory dilutions of cultured malaria parasites to create different malaria parasitaemia and haematocrit ratios.

### Haematocrit dilutions to mimic anaemic conditions

**Fig. 1:**
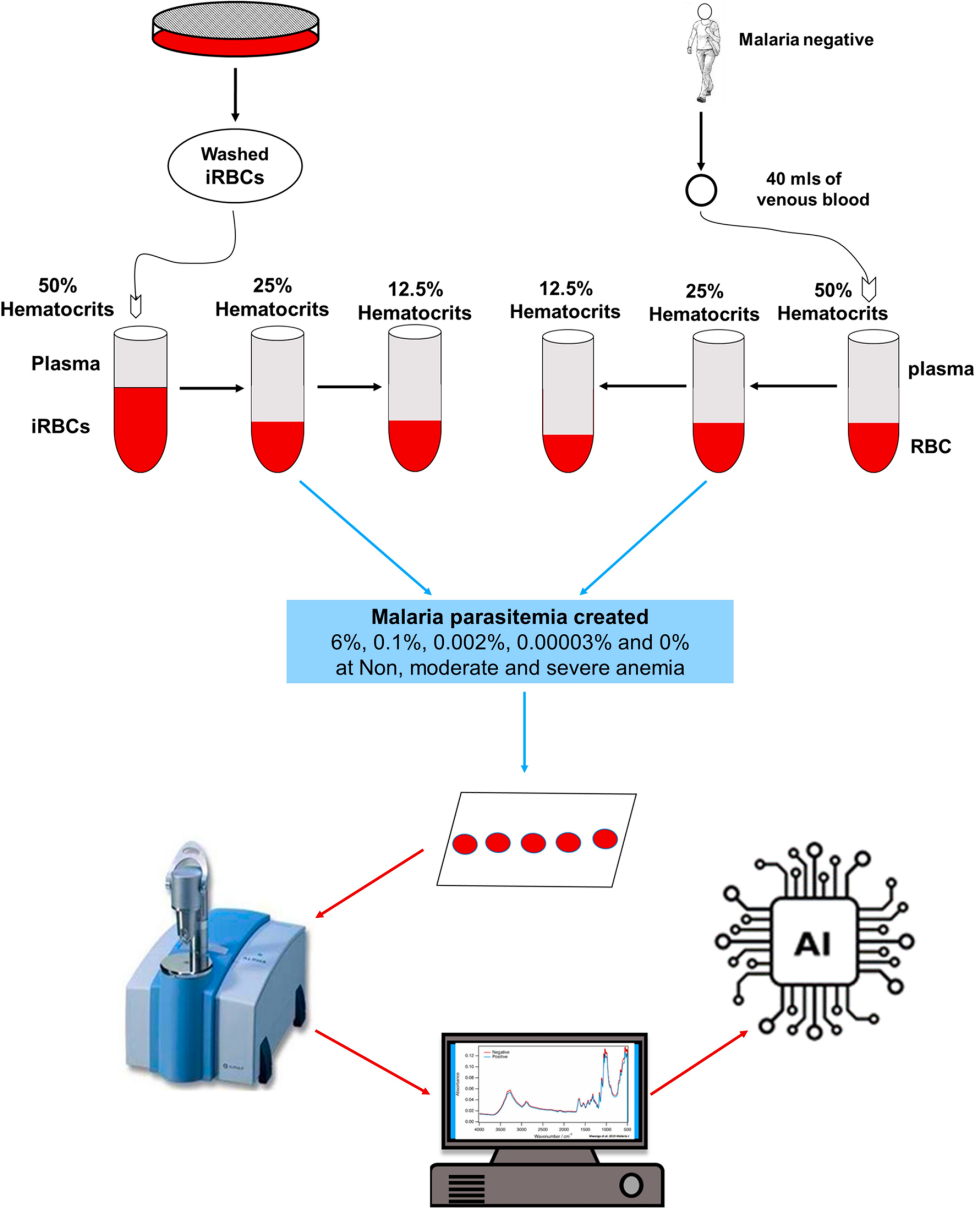
Schematic flow of experimental setup used to create DBS of different parasitaemia under non-anaemic (50%), moderate (25%), and severe anaemia (12.5%) and then scanned with MIR Spectrometer for spectra acquisitions and analysis using AI approaches

For each participant, two sets of haematocrit dilutions were created to simulate different anaemic conditions for both infected and un-infected blood. One set had malaria-free blood at 50%, 25%, and 12.5% haematocrit content, while the other comprised infected red blood cells (iRBCs) from cultured parasites, adjusted to 50%, 25%, and 12.5% haematocrit ratios using respective plasma. For uninfected blood, 40 ml of venous blood from each participant was split into 5 mL and 35 mL portions and centrifuged to separate plasma from RBCs. After separation, plasma was transferred to empty 1.5 mL tubes for haematocrit dilutions. RBCs from the 5 ml portion were used to formulate a 50% haematocrit stock solution by adding plasma from the same volunteer. Serial dilutions was done by transferring 2.5 mL of the stock solution to a second tube, and adding 2.5 mL of previously obtained plasma to simulate moderate anaemia (25% haematocrit) and severe anaemia (12.5% haematocrit) conditions (Fig. 1). On the other hand, for infected blood, when the culture reached > 6% ring stage parasitaemia, it was centrifuged to separate iRBCs from the culture media and washed twice. Washed iRBCs volume was 0.5 mL, which was mixed with an equal volume of participant plasma (0.5 mL) to create a 50% haematocrit ratio stock solution; and serially diluted to 25% and 12.5% solutions (Fig. 1).

### Serial dilutions for different parasite densities and controls

The parasite dilutions were performed at the respective haematocrit ratios. Initially, the cultured parasitaemia was standardized to 6% stock—and in cases where the initial densities were higher, this was lowered to the 6% densities. Serial dilutions were done to create three additional parasitaemia levels, i.e. 0.1%, 0.002% and 0.00003%, were created from the stock solution. The control group included malaria-negative samples at haematocrits of 50%, 25%, and 12.5% prepared from uninfected RBC from the culture with plasma from same individual participants. To ensure RBC distribution in control matched to that of malaria parasitaemia dilutions, uninfected haematocrits from the culture were diluted with uninfected haematocrits from participants in volumes equal to the parasitaemia dilutions (Fig. 1).

### Preparation of dried blood spots

For each individual volunteer, five replicates of dry blood spots (DBS) for each parasite density at each haematocrit level were created, resulting in a total of 75 DBS per participant. For each DBS, 50 μL of blood was added on the circular spot on the Whatman^™^ paper cards. The experimental design ensured that all malaria-positive and negative samples utilized similar filter paper cards to standardize the potential impact of filter paper on ML analysis. The cards were air-dried for up to 3 h and labelled with batch number, date, parasitaemia levels, haematocrit ratio, and participant ID. To prevent cross-contamination, each card was sealed in a plastic bag. The cards were then grouped by participant ID and stored in a cool environment in a larger bag with desiccant packets and a humidity card, awaiting transportation to another Ifakara Health Institute’s facility, the VectorSphere, at Ifakara, Tanzania for infrared scanning. During transport, the bags were kept in a cooler box with ice packs separated by plastic sheeting.

### Acquisition and pre-processing of infrared spectra

The DBS were individually scanned using a Fourier transform infrared spectrometer (FT-IR) with a wavenumber range of 4000–500 cm^−1^ and a resolution of 2 cm^−1^. The instrument employed was a compact Bruker Alpha FTIR interferometer, equipped with a Platinum-Attenuated Total Reflectance (ATR) module that incorporates diamond crystals. Scanning was done after 3–5 days of DBS storage. Each blood spot was punched and placed on the diamond crystal. For scanning, each blood spot was excised, positioned on the diamond crystal, and subjected to pressure using an anvil to enhance the contact area with the crystal, thereby optimizing the depth of light penetration. Each spot was scanned 32 times to obtain an averaged spectrum, which was labelled by project initial, study site, participant ID, haematocrit ratio, parasitaemia ratio, and dates.

To quantify the depth of light penetration (d) in whole blood, a theoretical approach that considers the wavelength of light (λ), incidence angle (θ), and the refractive indices of whole blood (n_1_) and the diamond crystal (n_2_) used in the spectrometer were employed. The penetration depth (d) (refer to Fig. S1) was calculated using the formula.

$${\text{D}} = \frac{{{\lambda n}_{1} }}{{2{\uppi }\sqrt {\sin^{2} {\uptheta } - \left( {\frac{{{\text{n}}_{2} }}{{{\text{n}}_{1} }}} \right)^{2} } }}$$

Given that the incidence angle (θ) was fixed at 45º, the refractive index of whole blood (n_1_) was determined using the Sellmeier equation (with λ expressed in micrometres) [31]:

$${\text{n}}1\left( {\uplambda } \right) = 1{ } + { }\left( {\frac{{0.7960 \times {\uplambda }^{2} }}{{{\uplambda }^{2} - 1.0772 \times 10^{4} }}} \right) + { }\left( {\frac{{5.1819 \times {\uplambda }^{2} }}{{{\uplambda }^{2} - 78301 \times 10^{5} }}} \right)$$

Similarly, the refractive index of the diamond crystal (n_2_) was ascertained through its corresponding Sellmeier equation [31]:

$${\text{n}}_{{2}} \left( \lambda \right) = 1{ } + { }\left( {\frac{{4.3356 \times \lambda^{2} }}{{\lambda^{2} - 0.1060^{2} }}} \right) + { }\left( {\frac{{0.3306 \times \lambda^{2} }}{{\lambda^{2} - 0.1750^{2} }}} \right)$$

The acquired spectra were then pre-processed using a Python program to compensate for atmospheric interferences, water vapour and carbon dioxide (CO_2_) and to discard spectra with poor quality as described by González-Jiménez et al. [18]. The pre-processed spectra were subsequently used for training, testing, and validating machine-learning algorithms.

### Selection of machine-learning models

Machine learning analysis was conducted using the Python programming language, version 3.9. Employing a supervised ML classification approach, seven classifiers were evaluated: Logistic Regression (LR), Support Vector Machine (SVM), Random Forest (RF), Gradient Boosting (XBG), Decision Tree (DT), Extra Tree (ET) Classifier, and Bagging Classifier (BC). The non-anaemic class (50% haematocrit) was utilized for ML algorithm selection and training, while the two other haematocrit ratios (25% and 12.5%) were kept separate and used to assess the impact of anaemia on the ability of the models to classify infected versus non-infected specimen from sets of previously unseen spectra. To do this, the non-anaemia data was shuffled and split into two portions; 70% for model selection, training, and testing, while 30% were kept separate as an unseen dataset for validating the trained model. Further, 70% portion were divided into 80–20% train-test split, respectively. For model selection, training, and testing, balanced classes were ensured through random under-sampling of the majority class.

Stratified shuffle split, tenfold cross-validation (SSS-CV) was employed to select the best machine-learning algorithm for identifying malaria infections. The seven mentioned algorithms were evaluated of the majority, and the best one was selected based on accuracy scores to distinguish malaria infections within the non-anaemia class using three approaches: (i) Cross-validation using datasets with high contrast (Positive class = 6%, N = 230) against the negative class (Negative = 0%, N = 230); (ii) Cross-validation with all concentrations (6%, 0.1%, 0.002%, and 0.00003%) combined as the positive class (N = 220) against the malaria-negative class (N = 220); and lastly, (iii) Cross-validation with low contrast (positive class = 0.00003%, N = 226) against the negative class (N = 226) datasets. Model selection, training and validation were performed on standardized absorption intensities relative to their wavenumbers.

### Training, testing and validation of machine learning models to identify malaria parasite presence in non-anaemic spiked blood

The best ML algorithms selected through SSS-CV were then trained on 80% of the spectra data from non-anaemic blood using three distinct approaches: (i) High Contrast: models were trained using highest parasite densities (6%) as positive samples against negatives (0%); (ii) All Concentrations: models were trained using combined all parasite densities (6%, 0.1%, 0.002% and 0.00003%) as positives, against negatives (0%); and (iii) Low Contrast: models were trained using lowest parasite densities (0.00003%) as positives, against negatives (0%). The trained models underwent fine-tuning through Grid Search for optimal hyper parameter optimization.

For testing purposes, the final tuned classifiers were tested on a similar parasitaemia class used for training. For instance, the model trained on 80% of the data with high contrast at 6% against negatives was also tested on the remaining 20% of the data at the highest contrast against negative classes. In addition to accuracy, other evaluation measures such as sensitivity, specificity, recall, and F1-score on the test set (20%) were calculated. Finally, the best classifiers were validated on a completely unseen dataset, the 30% kept separate at the start. Beginning with non-anaemic classes at different parasitaemia levels, the total number of DBS included for model validation in the non-anaemic class with various parasitaemia levels were as follows: validated model performance on predicting malaria infections at non-anaemic conditions when positive at 6% parasitaemia (N = 82) against negative 0% (N = 82); then validated positive at 0.1% (N = 82) against negative 0% (N = 82), followed by positive at 0.002% (N = 82) against negative 0% (N = 82), and finally, positive at 0.00003% parasitaemia (N = 82) against negative 0% (N = 82).

### Evaluating the effect of anaemia on performance of MIR-ML for distinguishing between blood samples with and without malaria parasites

The best classifiers developed for predicting malaria infections without considering anaemia were evaluated on a new dataset comprising cases of moderate anaemia (with a haematocrit level of 25%) and severe anaemia (with a haematocrit level of 12.5%). This evaluation was structured across four distinct categories: (i) malaria-positive with a 6% parasitaemia rate (N = 101) versus malaria-negative with a 0% parasitaemia rate (N = 101); (ii) malaria-positive with a 0.1% parasitaemia rate (N = 101) versus malaria-negative (N = 101); (iii) malaria-positive with a 0.002% parasitaemia rate (N = 101) versus malaria-negative (N = 101); and (iv) malaria-positive with a 0.00003% parasitaemia rate (N = 101) versus malaria-negative (N = 101). The accuracy, sensitivity, and specificity of these models computed using a bootstrap method with 100 iterations and establishing a 95% confidence interval for these metrics.

Finally, a generalized linear model was used to test the statistically significant effect of anaemia and parasitaemia on the performance of MIRs-ML approaches in predicting malaria infections.

## Results

### Generating samples of different parasite densities and anaemic conditions

In order to identify the lowest detectable concentrations of malaria parasites and assess the impact of anaemia on the predictive accuracy of MIRs-ML for malaria infections, cultured *P. falciparum* ring-stage parasites were diluted with malaria-negative blood from seventy volunteers to generate four different parasite densities (Fig. 1). The two lowest malaria parasitaemias (0.002% or 50–100 parasites/μL and 0.00003% or 1–3 parasites/μL) were selected to correspond to the approximate detection limits of RDT/microscopy and PCR, respectively. Additionally, parasitaemia levels of 6% and 0.1% were chosen to capture the highest parasite contrasts in the training dataset. Haematocrit was set at 50%, 25%, and 12.5% to represent normal, moderate, and severe anaemia, respectively. Thus, a two-way matrix consisting of four malaria-positive parasitaemia levels (6%, 0.1%, 0.002%, and 0.00003%) and a negative class (0%), along with three anaemic classes, was generated (Table S1). In total, 4655 DBS were created and scanned using MIR spectrometer, which were used to train and evaluate ML classifiers. Samples were randomly selected to confirm the success of dilutions (Figs. S2 and S3).

### Selection and training of machine-learning classifiers

4559 spectra were analysed with varying malaria parasite densities and haematocrit ratios. Of these, 12 spectra from the non-anaemic samples, 35 from moderately anaemic samples, and 49 from severely anaemic samples were discarded due to excessive water content resulting from plasma and atmospheric interference (Table S2). The mean spectra of each parasitaemia class at different anaemic conditions revealed characteristic biochemical signatures of *P. falciparum* infections such as amide, lipids, and haemozoin (Figure S1) [32]. To ensure ML classifiers would learn features associated with malaria infections, an infrared region without the key biochemical information (from 2799 to 1801 cm^−1^) was eliminated [21], and the regions 3100–2800 cm^−1^ and 1800–900 cm^−1^ were selected to train and validate ML classifiers.

**Fig. 2:**
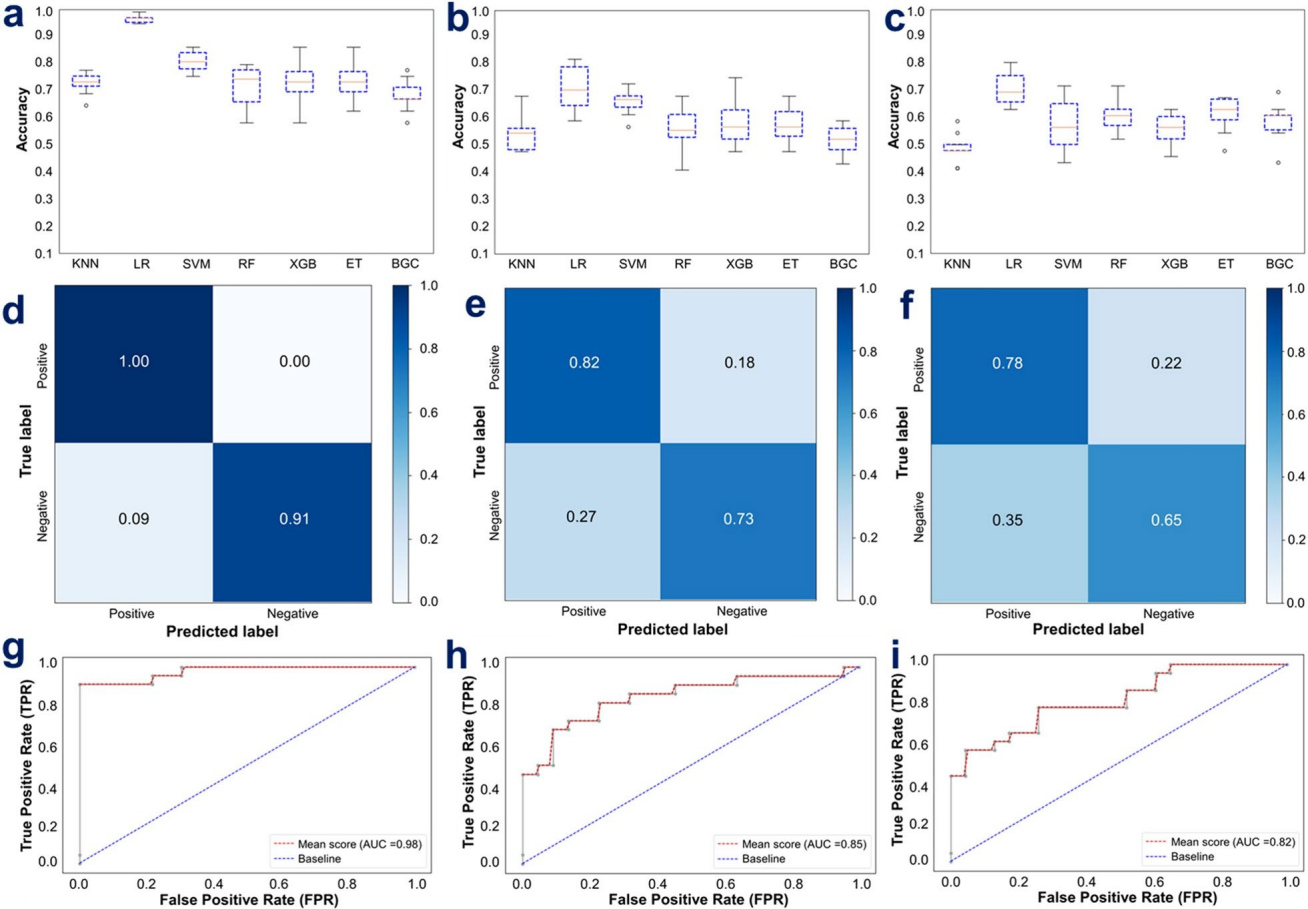
The performance of the seven ML classifiers assessed through cross-validation on non-anaemic samples, following three approaches. **a** Accuracy scores of classifiers in high contrast against the negative class. **b** Evaluation with all concentrations, combining all parasite densities as positive against none. **c** Assessment in low contrast against the negative. Confusion matrices for trained and fine-tuned LR models on the 20% test set, with parasitaemia class similar to the training set, are displayed in panels **d**, **e**, and **f**. Receiver Operating Characteristics (ROC) and Area Under the Curve (AUC) of three tuned LR models are presented for high contrast (**g**), all concentrations (**h**), and low contrast (**i**)

First, using tenfold stratified shuffle split cross-validation (SSS-CV), LR achieved the highest SSS-CV accuracy score in all three datasets, achieving 97.61%, 71.14% and 70.87% in high contrast, all concentrations combined and low contrast training sets, respectively (Fig. 2a–c).

LR algorithm was retained for further tuning, utilizing a grid search approach over an extensive range of hyperparameter values. The tuned model, when fitted to high-contrast scenarios characterized by a 6% parasitaemia level versus non-infected samples, demonstrated remarkable capacity to distinguish malaria-positive and -negative samples, achieving accuracies of 100% and 91%, respectively (Fig. 2d). When evaluating the performance of the tuned models across all parasitaemia concentrations combined, the accuracy rates for correctly identifying positive and negative samples were 82% and 73%, respectively (Fig. 2e). Even in the context of low-contrast model, trained with the 0.0003% parasitaemia against negative, the model maintained strong performance, with positive and negative detection accuracies of 78% and 65%, respectively (Fig. 2f).

Even after tuning the LR to optimize its hyperpartemeter, training with high contrast had the largest area under the curve (AUC) estimate, 0.98 (Fig. 2g), outperforming models trained using both the combined concentrations and low contrast data sets, for which the AUC scores were 0.85 and 0.82, respectively (Fig. 2h, i). The train-test size, precision, recall, and F1-score of the three models on the validation set are summarized in Table 1.

**Table 1:** Summarized scores (Precision, Recall, and F1-score) for testing the three LR models on the 20% train-test splits using laboratory data

Sample size		Precision score (%)		Recall score (%)	F1-score (%)
Classifier 01: trained on high contrast (6%) against 0%					
Train size	460	Positive class	92	100	96
Test size	92	Negative class	100	91	95
Classifier 02: trained on all concentrations (6%, 0.1%, 0.002%, 0.00003%) against 0%					
Train size	440	Positive class	75	82	78
Test size	88	Negative class	80	73	76
Classifier 03: trained on low contrast (0.00003%) against (0%)					
Train size	452	Positive class	69	78	73
Test size	91	Negative class	75	65	70

**Fig. 3:**
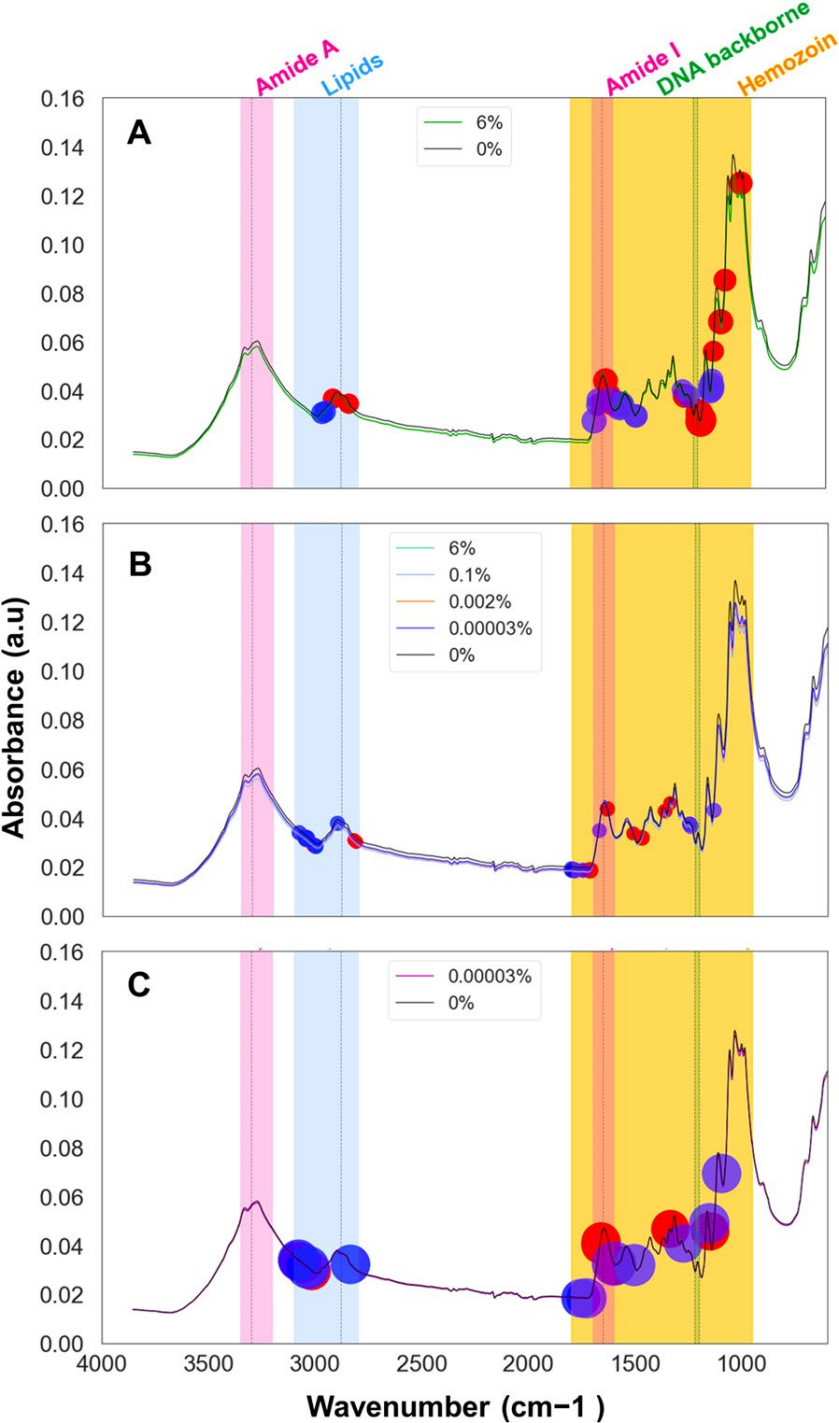
Spectral features with the greatest influence on the performance of the three models, **a** High contrast, **b** All concentrations combined, and **c** Low contrast training sets for the prediction of positive class (Red circle) and negative class (Blue circle). The size of the circle represents coefficient scores

In addition, to understand the specific biochemical contribution influencing predictions, the wavenumber values were extracted with corresponding coefficients that most influenced the performance of three trained LR classifiers. All three classifiers have learned from wavenumber values associated with biochemical signals associated primarily with lipids (3100–2800 cm^−1^) and proteins (1800–600 cm^−1^) (Fig. 3a–c), consistent with signals expected from malaria infections [16, 21].

The highest contrast model appears to have primarily learned to classify malaria infections from the wavenumbers associated with proteins, notably amide III and amide I vibrations (1185–1191 cm^−1^, 1199–1201 cm^−1^, 1619–1617 cm^−1^ and 1687–1671 cm^−1^), which are indicative of the secondary structure of proteins. Wavenumber values 997, 1125–1139 cm^−1^ highlight the models’ use of C-N stretching vibrations of proteins (peptides) and phosphodiester stretching (1201, 1071, and 1263 cm^−1^) indicative of nucleic acids (Fig. 3A). The high-contrast model effectively identified wavenumbers linked to lipids, alkanes, and carbohydrates, indicated by key C–H stretching vibrations at 2917 cm^−1^, 2843 cm^−1^, 2967 cm^−1^, and 2955 cm^−1^, and C–H bending vibrations at 1491 cm^−1^ and 1493 cm^−1^. Optimal wavenumber values for the other LR models, covering all concentrations and low-contrast scenarios, are also visually indicated in Fig. 3B, C.

**Fig. 4:**
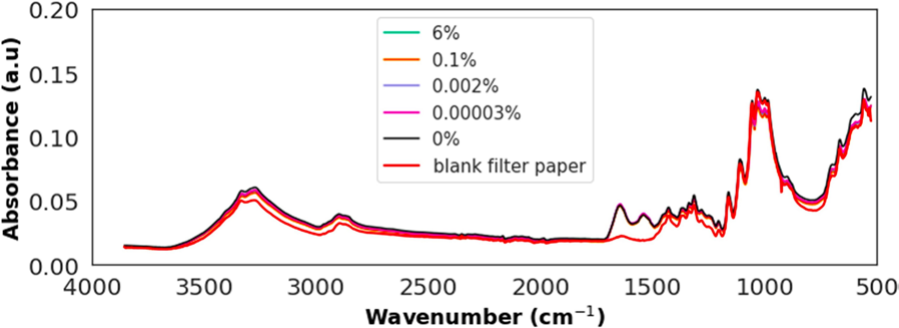
Visual comparison of mid-infrared spectra from blank filter paper with those containing varying levels of malaria parasitaemia in malaria-positive blood (6%, 0.1%, 0.002%, and 0.00003%) and malaria-negative blood (0%). The figure represents averaged spectra from 10 filter papers, each scanned 32 times, i.e. 320 spectral scans

Visualization of blank filters paper revealed distinct peaks in the wavenumbers between 1700 and 1200 cm^−1^ in both malaria-positive and malaria-negative blood samples (Fig. 4), where most of the features of importance for all three classifiers are concentrated (Fig. 3A–C). Additionally, a similar spectral trend was observed in the 1100–500 cm^−1^ range across the blank filter paper and both malaria-positive and malaria-negative samples (Fig. 4). However, of the 40 key features identified by the classifier trained with high contrast samples (Fig. 3A), only 6 fell within this overlapping region, with 4 out of those 6 having positive coefficients. The other two classifiers—one trained on all concentrations and the other trained with low contrast against negative samples—had fewer features originating from this region (Fig. 3B, C). This suggests that cellulose, a component of the filter paper, did not significantly affect the accuracy of the classifiers.

### Detection of malaria infections at different parasitaemia and anaemic conditions

After establishing that the high contrast training set had the best performance in predicting a dataset of the same parasitaemia levels, the accuracy of the three trained LR algorithms was validated against various parasitaemia and anaemic conditions using 100 bootstrap random resampling. For this purpose, 30% of the full dataset was held out separately before training as unseen data.

**Fig. 5:**
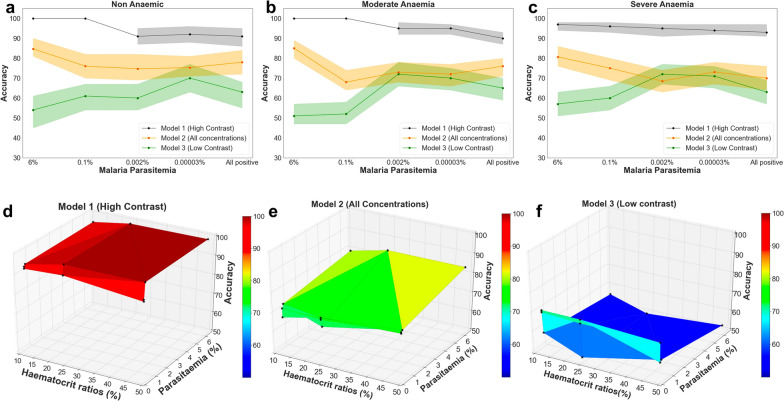
Performance of three LR models on a completely unseen dataset held out prior to training, for non-anaemic (**a**), moderate (**b**), and severe anaemia (**c**). **d**–**f** represent the three-dimensional representation of the LR performance on the validation set for high contrast, all concentrations, and low contrast models, respectively

The MIRs-ML trained on the high contrast data set against the negative class identified malaria infections in non-anaemic samples with 100% accuracy for the two highest parasitaemia levels tested (6% and 0.1%). Accuracy dropped to 92–91% when classifying intermediate and low-level parasitaemia, and when evaluating combined spectra from all concentrations (Fig. 5a). Similarly, high accuracies were observed when the high-contrast model was used to predict parasite infections at moderate and severe anaemia (Fig. 5b, c).

Models trained on all combined concentrations and those trained using low contrast were also validated for predicting various parasitaemia in an unseen dataset. Regardless of anaemia, MIRs-ML trained on all concentrations accurately classified different parasitaemia, achieving an average accuracy of 75.33% [70.07–80.8%] (Fig. 5a–c). The model trained on the lowest contrast demonstrated an average accuracy of 62.73% [56.53–74.00%] across different parasitaemia and anaemia. When validating the low-contrast model on different parasitaemia and anaemic conditions in new data, an increased accuracy was noticed in predicting parasitaemia levels resembling those in the training set (moderate and low parasitaemia) (Fig. 5a–c).

Overall, the high-contrast model outperformed the other two models in predicting malaria infections at all tested parasitaemia and anaemic conditions (Fig. 5d–f), suggesting that this approach is the most robust for achieving generalizability.

A generalized linear model was fitted to estimate the impact of training methodologies, levels of parasitaemia, and the presence of anaemia on the predictive accuracy of MIRs-ML in diagnosing malaria infections. The analysis revealed that neither anaemia nor the different levels of parasitaemia significantly affected the capabilities of the MIRs-ML models for distinguishing between infected and non-infected samples (anaemic conditions: χ^2^ = 0.01, p = 0.99; parasite intensities: χ^2^ = 0.24, p = 0.99). However, the choice of training methodology significantly affected model performance (χ^2^ = 201.62, p < 0.001). Further analysis using *Post-Hoc* Tukey's test to compare the three training methodologies showed that training with high-contrast samples notably enhanced the predictive accuracy of MIRs-ML, yielding a mean difference of 19.93% (p < 0.001) compared to training with all concentrations, and a mean difference of 32.53% (p < 0.001) compared to training with low-contrast samples. Additionally, training with all concentrations demonstrated a significant positive effect, with a mean difference of 12.59% (p < 0.001), when compared to training with low-contrast samples.

### Validation of the MIRs-ML for identifying malaria infections using spectra from field-collected dry blood spots

**Fig. 6:**
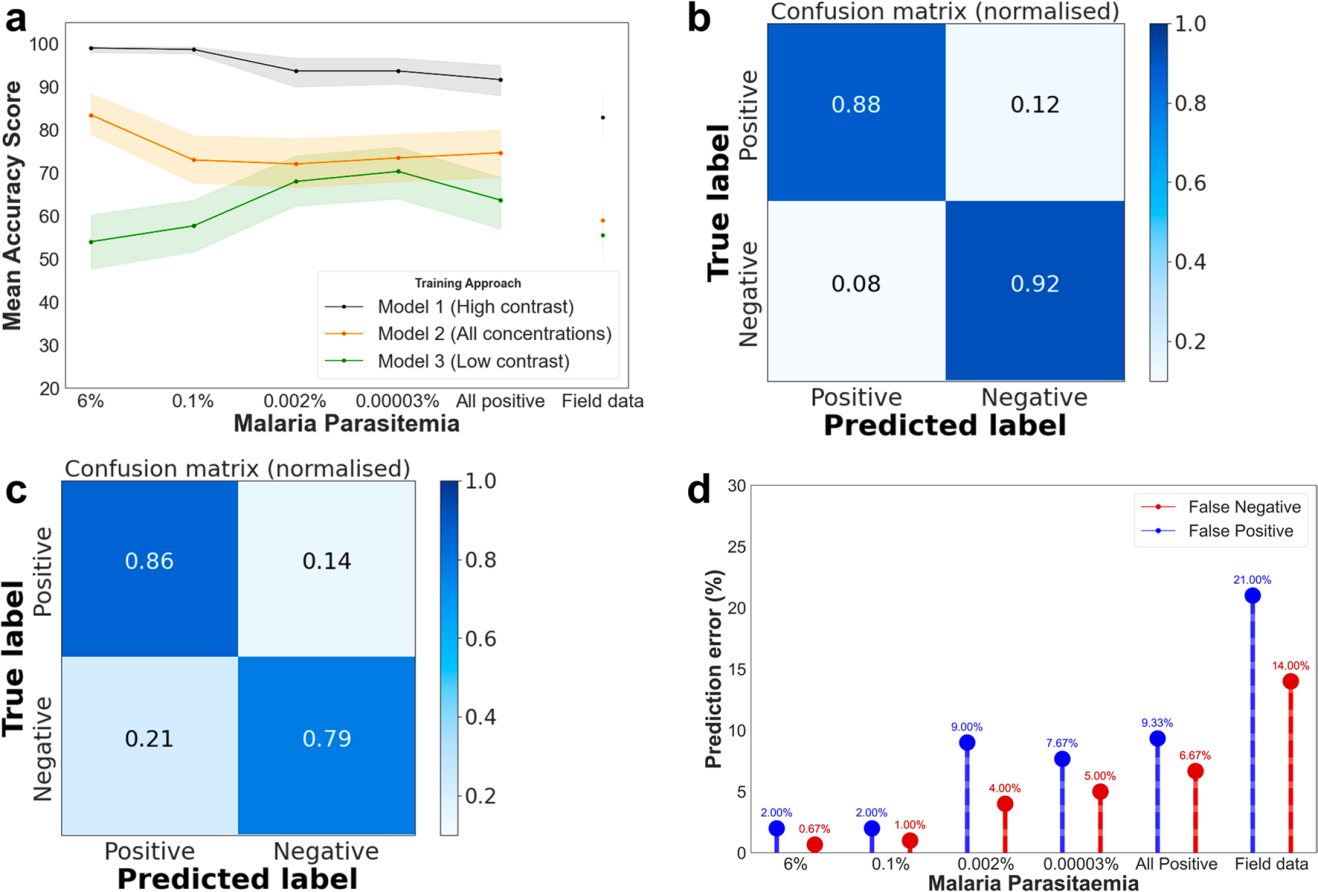
Evaluation of three trained logistic regression (LR) models for classifying malaria infections in laboratory and patient samples. **a** Mean accuracy for each anaemia level across various parasitaemia levels using laboratory and field-collected DBS. **b** Confusion matrix of the high-contrast model predicting laboratory-combined parasitaemia against negative in non-anaemic conditions, simulating realistic field collections. **c** Confusion matrix indicated the high-contrast model’s performance in detecting malaria infections in realistic field-collected DBS. **d** False positive and false negative predictions by the high-contrast model averaged by anaemic conditions using both laboratory and field samples

The performance of laboratory-trained models was evaluated using realistic samples collected from patients. To facilitate comparison, the created models were tested under two scenarios. Firstly, by employing laboratory data as detailed in the preceding sections. Secondly, by utilizing a realistic dataset obtained from the field (see Fig. 6a). In simulations resembling field conditions, where all parasitaemia levels generated in the laboratory were combined and treated as positive, the high-contrast trained model distinguished positive from negative samples with accuracies of 88% and 92%, respectively (Fig. 6b).

Next, 252 DBS samples collected from a previous field survey in southeastern Tanzania were scanned [16]. The malaria infection status in these samples had been confirmed using nested PCR [16]. In these tests, the model with the highest contrast predicted malaria infections with an accuracy of 83%, achieving 86% accuracy for the positive class and 79% accuracy for the negative class (refer to Fig. 6c). The precision, recall, and F1-score of the three trained models when tested with these field-collected dataset are summarized in Table 2.

**Table 2:** Summarized scores (Precision, Recall, and F1-score) for validating the three LR models on field-collected samples

	Precision score (%)	Recall score (%)	F1-Score (%)
Classifier 01: trained on high contrast (6%) against 0%			
Positive class	84	86	85
Negativeclass	81	79	80
Classifier 02: trained on combined positive (6%, 0.1%, 0.002%, 0.00003%) against 0%			
Positive class	58	59	58
Negative class	51	50	50
Classifier 03: trained on lowest parasitaemia (0.00003%) against (0%)			
Positive class	57	58	57
Negative class	57	56	57

To understand the potential applications of MIRs-ML for either point-of-care or population surveys, false negatives and false positives of the highest contrast model were computed in predicting all parasitaemia in laboratory specimens and field datasets (Fig. 6d). The model exhibited a prediction error of 14% for false negatives and 21% for false positives in field samples, preliminary suggesting that this approach could be most useful in population surveys, especially in low transmission settings, due to the lower rate of false negatives.

## Discussion

Effective malaria screening is crucial for guiding elimination efforts, especially in detecting low-density parasitaemia. However, current methods often face trade-offs between cost and sensitivity. Alternatively, infrared spectroscopy coupled with machine-learning (MIR-ML) is showing great promise, particularly in laboratory settings. Here, the findings suggest that the MIR-ML approach can detect as few as 1–3 parasites/μL of blood with an overall accuracy exceeding 90% under laboratory settings even with presence of anaemia. Furthermore, this study as the first, demonstrate that ML algorithms trained with the highest plausible parasite concentrations against negative samples (“high contrast” training set) can yield a potent and robust classifier capable of predicting malaria infections at various parasitaemia levels, regardless of the presence of anemia. Although anemia is highly correlated with malaria infections, this study reports for the first time that it does not impact the performance of the ML approach in predicting malaria infections. Notably, the ML model trained with laboratory-generated high contrast dataset accurately classified over 80% of field-collected specimens obtained from malaria-infected patients. Since these specimens were entirely unseen by the model, this indicates good generalizability of this approach and holds great promise for its future optimization in malaria detection, especially for field surveys.

This study demonstrates the ability of MIRs-ML to detect low parasite concentrations (1–3 parasites/μL), which are often missed by methods such as microscopy and RDTs [33, 34], the latter of which is also compromised by HRPII gene deletions [35, 36]. Additionally, this study improves upon earlier studies that applied multivariate analysis, including partial least squares and classical regression approaches, to detect and quantify malaria infections in isolated infected and uninfected RBCs by revealing that the integration of MIRs-ML can detect as low as 1 parasite/μL of blood with an accuracy exceeding 90% in DBS, even in the presence of anaemia, and requiring minimal sample handling.

While malaria is the primary cause of anaemia in regions with high transmission risks, other infections, including gastrointestinal helminths, can also cause anaemia, especially in children and the elderly [37–39]. If MIRs-ML detects anaemia (e.g. where anaemia may be confounded with malaria presence), there could be a risk of misdiagnosis disproportionality affecting children, who are more vulnerable to the effects of misdiagnosed malaria [24]. Therefore, experimentally anaemia levels were simulated by adjusting plasma to RBC ratios using blood from volunteers. MIRs-ML trained on the high contrast dataset predicted malaria infections in the presence of severe anaemia, with accuracy exceeding 90% even at the lowest parasitaemia, indicating that the predictive capabilities of IR-ML are not affected by anaemia.

A further potential problem for IR-ML technologies is poor generalizability to new datasets, as observed in entomological traits of malaria vectors due to genetic, environmental, and dietary variation [15, 19]. Previous studies involving the training of IR approaches for malaria detection used low contrast or less immunologically diverse blood to spike with parasite culture [15]. Here, models were trained on blood samples from 70 individuals to capture population immunological variability, experimentally spiked with cultured ring-stage *P. falciparum* parasites. This allowed models to achieve over 80% accuracy in diagnosing malaria infections with field data without further calibrations. To further improve generalizability, techniques, such as transfer and partial learning [19, 40] could be useful for improving the predictive accuracy of this approach, especially in diverse population surveys.

The trained algorithms in this study learned from peaks associated with the biochemical signatures of malaria-infected samples, particularly those suggested to be linked with the by-products of the malaria ring-stage parasite, such as lipids, proteins (haemozoin) [32], and parasite DNA. In this study, the blood sample was prepared by removing the buffy coat and white blood cells by suction when separating plasma from RBCs for anaemic adjustment. Therefore, the host cells present in the sample were RBCs, which are enucleated. Thus, the signal observed corresponding to phosphodiester stretching (997, 1201, 1071, and 1263 cm^−1^) are likely due to parasite nucleic acids only. Further, differences in the peaks related to the amide groups of the proteins were identified. Specifically, the band between 3500 and 3000 cm^−1^, commonly referred to as Amide A and associated with the stretching of N–H bonds; the band between 1700 and 1600 cm^−1^ (Amide I), primarily linked to the C=O stretching vibration and sensitive to the secondary structure of the protein; the band between 1600 and 1500 cm^−1^ (Amide II), mainly due to N–H in-plane bending and C–N stretching vibrations; and the band between 1350 and 1200 cm^−1^ (Amide III), which is a combination of C–N stretching, N–H in-plane bending, and C–H in-plane bending vibrations, appear crucial in capturing the variations due to malaria infections. Also changes in the peaks associated with the C–H stretching modes of the lipids (2901, 2905, and 2859 cm^−1^) were observed.

Analysis of blank filter paper, compared to filter paper containing malaria-positive and -negative samples, indicated that the filter paper was adequately controlled for and did not affect the model performance. This is consistent with other reports that identified significant bands in the fingerprint region, which correspond to the presence of malaria parasites in blood samples [21, 22, 32, 41]. Spectral analysis of human blood has also revealed key features in the cellulose-overlapping region of 1100–500 cm^−1^, providing additional insights into distinguishing blood samples [42–46]. For example, Mistek-Morabito et al. used the wavenumber ranges of 4000–2800 cm^−1^ and 1800–600 cm^−1^ to differentiate human blood from other organisms, emphasizing the importance of the fingerprint region (below 1500 cm^−1^) [47]. Given the well-controlled experimental design of this study, which ensured standardization in the use of filter papers for both malaria-negative and malaria-positive samples, it is likely that the features observed in the fingerprint region offer valuable information for detecting malaria parasites, considering the characteristic peaks in human blood.

The advantage of using MIRs-ML for malaria parasite detection relies on its higher sensitivity and accuracy cost ratio, demonstrating potential applicability in low-income communities [15]. Standard low-cost maintenance, such as providing desiccants to limit humidity effects, is the only requirement for operation, with no need for additional reagents. This study demonstrated an added advantage of the approach, its capability to detect as low as one parasite/μL of blood in the presence and absence of anaemia, highlighting the potential use-case of MIRs-ML for malaria parasite detection in field settings. This technology has potential to be an easy-to-use method for screening parasite infections, and requires minimal training, specifically in sample handling, spectra acquisition, and result interpretation. This reduced training requirement is a major advantage over technologies like microscopy and PCR, which require high levels of expertise. This study, along with ongoing efforts to develop portable surveillance tools using similar technology [48, 49], suggests that this approach could be scalable, and might in future be integrated into routine health facilities, or even adapted for population surveys in rural areas. Bench-top devices range in cost from $20,000 to $60,000 but are reagent-free and require minimal maintenance, with a lifespan exceeding 10 years. This operational cost advantage, combined with the sensitivity demonstrated in this study, makes them a viable alternative to more expensive PCR-based technologies. Even RDTs, which are more affordable, tend to have lower sensitivity and may be particularly less reliable in low-transmission settings [15]. Additionally, there are now pocket-sized devices, which can cost as little as $2000, which offer opportunities for non-invasive diagnostics through the skin, allowing for fast, real-time results for malaria [48]. However, to better integrate the methods, further investigations are needed to understand full needs [15].

One important question to consider is where MIRs-ML should be utilized, either as a point-of-care test or for population surveys. Using a field-collected dataset, this study revealed that MIRs-ML algorithms detected unseen field-collected specimens with a lower false-negative rate of 14% compared to a false-positive rate of 21%. This might primarily indicate a potential use case of MIRs-ML for population surveys that could complement PCR confirmation of positive MIRs-ML samples. However, these should be considered as preliminary observations and for the further integration of MIRs-ML into routine malaria surveillance, large-scale clinical trials are essential to validate the feasibility of MIRs-ML for malaria parasite detection in vertebrate hosts.

This study solely focused on detecting the *P. falciparum* parasite, prevalent in endemic regions. It is crucial to explore detection limits and infrared signals associated with infections from *Plasmodium ovale*, *Plasmodium vivax*, and *Plasmodium malariae*. Future studies may also explore the performance of MIRs-ML in detecting malaria infections across various epidemiological strata, as the current tools exhibit different performance characteristics based on transmission patterns, with RDTs and microscopy showing poor performance in low-transmission areas. Moreover, the infrastructure requirements for IR-ML, including hardware and software configurations, training needs, and maintenance levels, should be addressed [15].

Additionally, it is important to further investigate malaria diagnostic interference of MIRs-ML due to other potential confounding factors, such as coinfection and nutritional factors.

## Conclusion

To advance malaria elimination, there is a critical need for screening methods that combine cost-effectiveness with the ability to detect low-density parasitaemia, a gap not filled by current methods like RDTs, microscopy, or expensive PCR. This study demonstrated an AI-driven mid-infrared spectroscopy technique that excels in identifying malaria infections in dried blood spots, achieving high accuracy across various parasite densities. The detectable limit of malaria parasite by MIRs-ML is below the lowest concentration tested, which is ~ 1 parasite per microlitre of blood. The performance of the technique was not compromised by anaemia, which is a frequent complication in malaria patients. Moreover, when MIRs-ML models initially trained on laboratory data were used to distinguish between infected and uninfected field samples collected from a field survey in rural Tanzania, the classification accuracies were maintained above 80%. These findings indicate the significant potential and viability of this AI-driven mid-infrared spectroscopy technique as an affordable and malaria-scalable screening tool in low-resource settings. Further validation in diverse areas and the consideration of additional confounders, such as co-infections, are necessary to further validate the approach in real-life settings.

### Supplementary Information

Supplementary material 1.

## Data Availability

The dataset supporting the findings is available upon a reasonable request, which can be directed to either the corresponding author, the Ifakara Health Institute ethical review board in Tanzania or National Health Research Ethics Committee in Tanzania with reference to ethical clearance certificate of NIMR/HQ/R.8a/Vol. 1X/3735.

## Abbreviations

AI: Artificial intelligence
MIRs: Mid-infrared spectroscopy
ML: Machine learning
ATR-FTIR: Attenuated total reflection-Fourier transform infrared
RDTs: Rapid diagnostic tests
PCR: Polymerase chain reactions
ELISA: Enzyme-linked immunosorbent assays
LAMP: Loop-mediated isothermal amplification
DBS: Dried blood spots
EDTA: Ethylenediaminetetraacetic acid
RPMI: Roswell Park Memorial Institute 1640 media
HEPES: 4-(2-Hydroxyethyl)-1-piperazineethanesulfonic
LR: Logistic Regression
SVM: Support Vector Machin
RF: Random Forest
XGB: Gradient Boosting
DT: Decision Tree
ET: Extra Tree
BC: Bagging Classifier

## Code availability

All code and their respective output for machine learning and statistical analysis are available at https://github.com/Issamshani17/MIRs-ML-Limit_of_detection
