# Supplementary material for: Screening of malaria infections in human blood samples with varying parasite densities and anaemic conditions using AI-Powered mid-infrared spectroscopy
Source: Malar J. 2024 Jun 17;23:188. doi: 10.1186/s12936-024-05011-z (PMC11181574; doi:10.1186/s12936-024-05011-z)
Supplement: Supplementary file 1 — Supplementary material 1. [file 12936_2024_5011_MOESM1_ESM.doc]

**Screening of malaria infections in human blood with varying parasite densities and anaemic conditions using AI-Powered mid-infrared spectroscopy**

Issa H. Mshani^1, 2^*, Frank M. Jackson^1^, Rehema Y. Mwanga^1^, Prisca A. Kweyamba^1,3,4^, Emmanuel P. Mwanga^1, 2^ , Mgeni M. Tambwe^1^, Lorenz M. Hofer^1,3^, Doreen J. Siria^1,2^, Mario González-Jiménez^2, 5^, Klaas Wynne^5^, Sarah J. Moore^1,3,4,6^, Fredros Okumu^1, 2, 6, 7†^, Simon A. Babayan^2†^ and Francesco Baldini^1,2†^

1. Ifakara Health Institute, Environmental Health, and Ecological Sciences Department, Morogoro, United Republic of Tanzania.
2. The University of Glasgow, School of Biodiversity, One Health and Veterinary Medicine, Glasgow, UK.
3. Swiss Tropical and Public Health Institute, Kreuzstrasse , 4123 Allschwil, Switzerland
4. University of Basel, Petersplatz , 4001 Basel, Switzerland
5. The University of Glasgow, School of Chemistry, Glasgow G128QQ, UK.
6. Nelson Mandela African Institution of Science and Technology, School of Life Sciences and Biotechnology, Arusha, United Republic of Tanzania.
7. The University of the Witwatersrand, School of Public Health, Park Town, South Africa.

^†^Simon A. Babayan, Fredros Okumu and Francesco Baldini are equally co-supervised this work.

*Correspondence:

1. Issa H. Mshani: [imshani@ihi.or.tz](mailto:imshani@ihi.or.tz)

2. Francesco Baldini: [Francesco.Baldini@glasgow.ac.uk](mailto:Francesco.Baldini@glasgow.ac.uk)

3. Simon A. Babayan: [Simon.Babayan@glasgow.ac.uk](mailto:Simon.Babayan@glasgow.ac.uk)

4. Fredros Okumu: [fredros@ihi.or.tz](mailto:fredros@ihi.or.tz)

**Table S1:** A two-way matrix of dried blood spots (DBS) generated in laboratory and used to train, test and validate machine-learning classifiers.

| Hematocrit concentrations that mimic anemic condition | | | | | |
| --- | --- | --- | --- | --- | --- |
| Malaria parasitemia (%) |  | Normal  (40-50%) | Moderate anemia  (25%) | Severe anemia (12.5%) | **Total** |
|  | 6% | 335 | 335 | 335 | 1005 |
|  | 0.1% | 335 | 335 | 335 | 1005 |
|  | 0.002% | 335 | 335 | 335 | 1005 |
|  | 0.00003% | 335 | 335 | 335 | 1005 |
|  | 0% | 335 | 150 | 150 | 635 |
| Total DBS | | 1675 | 1490 | 1490 | **4655** |

**Table S2:**  A summary of total spectra discarded due to either excessive water content, atmospheric water vapor and carbon dioxide interferences or bad intensity.

| Hematocrit concentrations that mimic anemic condition | | | | | |
| --- | --- | --- | --- | --- | --- |
| Malaria parasitemia (%) |  | Normal  (40-50%) | Moderate anemia  (25%) | Severe anemia (12.5%) | **Total** |
|  | 6% | 4 | 5 | 11 | 20 |
|  | 0.1% | 2 | 10 | 8 | 20 |
|  | 0.002% | 1 | 6 | 16 | 23 |
|  | 0.00003% | 2 | 3 | 6 | 11 |
|  | 0% | 3 | 11 | 7 | 22 |
| Total DBS | | 12 | 35 | 49 | **96** |


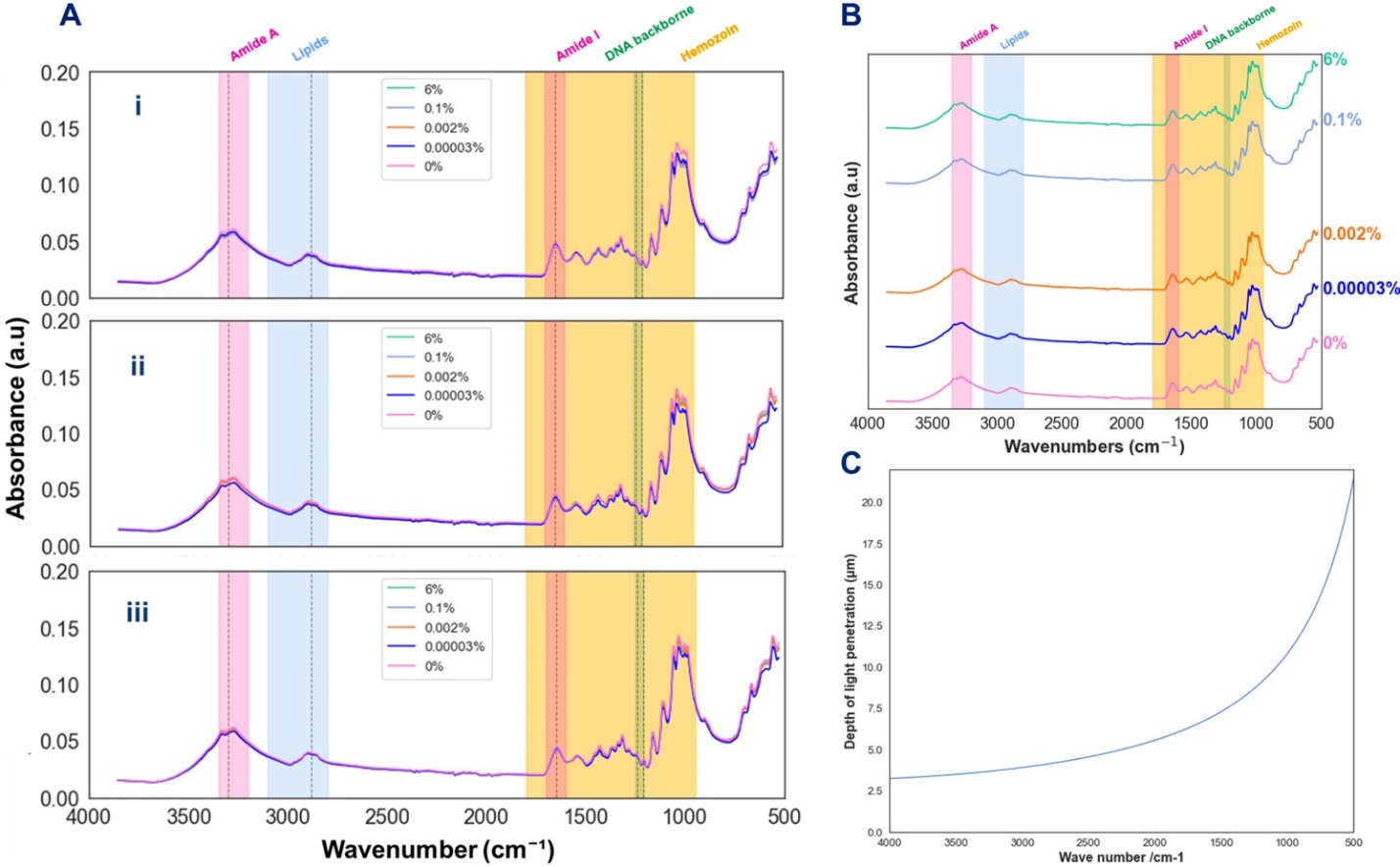


**Figure S1:** Average spectra of the generated anemic conditions, non-anemic (Ai), moderate (Aii), and severe anemia (Aiii) spectra. (B) Illustrates the magnified (Ai) for a better visualization of the average spectra of specific parasitemia in non-anemic samples. (C) Represent the estimated depth of light penetration to the DBS sample.


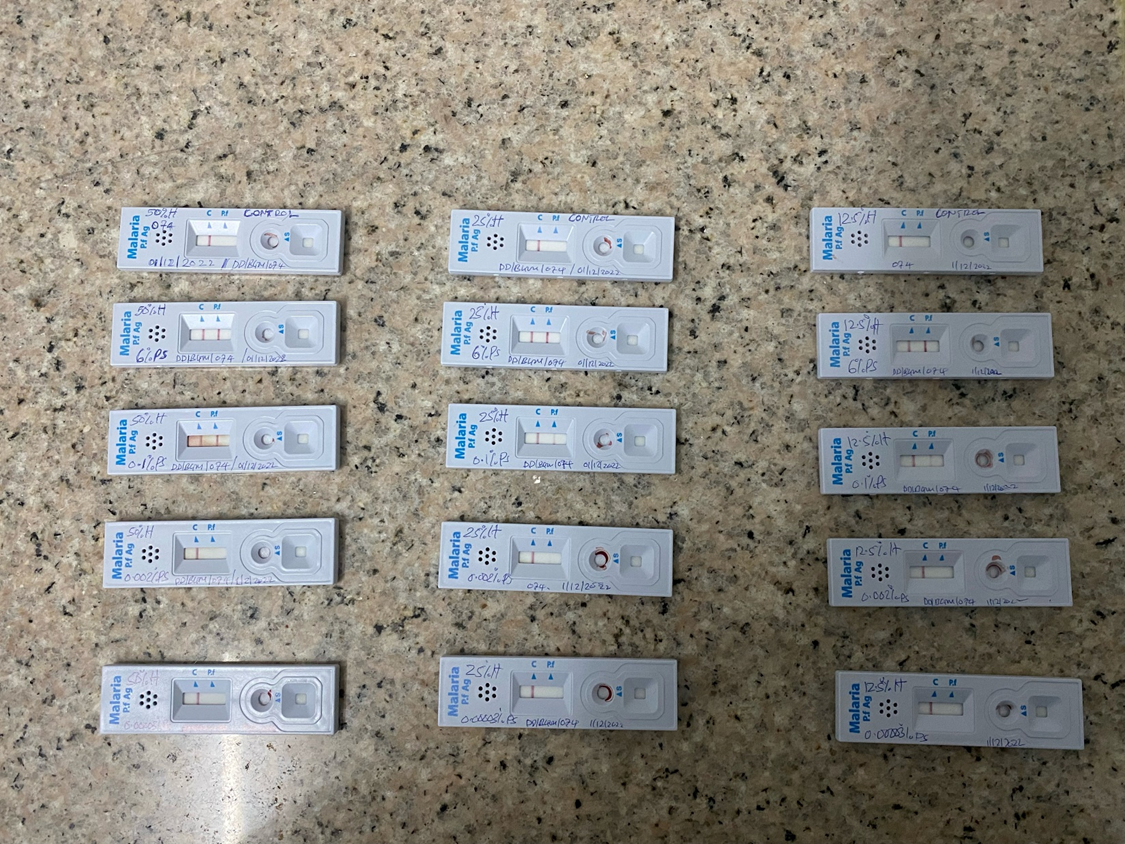


**Figure S2:** RDTS showing results when we tested the final dilutions sample as part of quality assurance processes


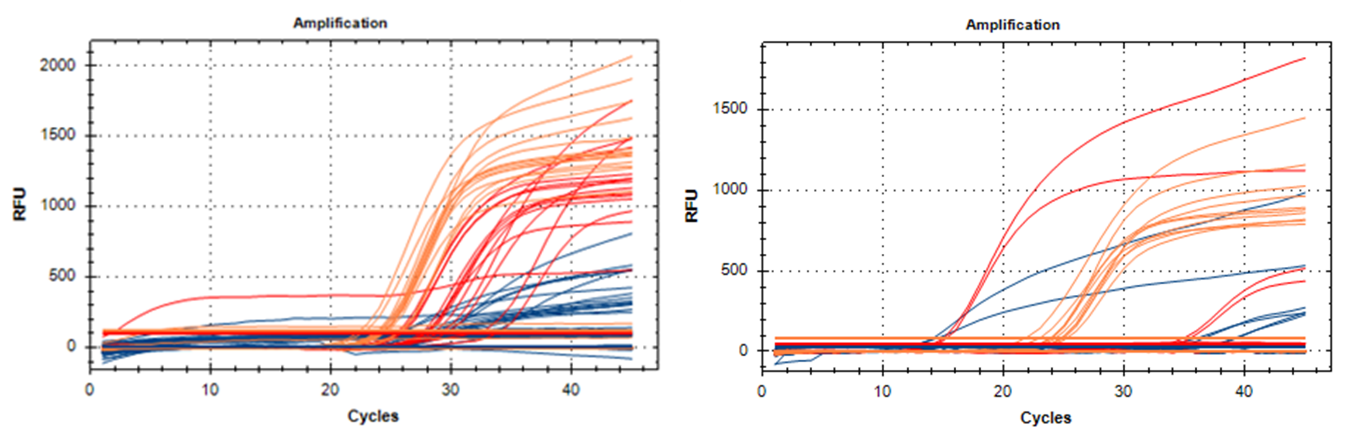


**Figure S3:** A representative PCR amplification cycles for the random samples generated in the laboratory as part of quality assurance processes
